# Analytical techniques for arsenic speciation

**DOI:** 10.1007/s44211-025-00722-y

**Published:** 2025-01-31

**Authors:** Yu-ki Tanaka, Kemmu Matsuhashi, Yasumitsu Ogra

**Affiliations:** Graduate School of Pharmaceutical Sciences, Chiba University, 1-8-1 Inohana, Chuo, Chiba 260-8675 Japan

**Keywords:** Arsenic, Speciation, LC-ICP-MS, GC-MS

## Abstract

Due to its toxicity, contamination with arsenic, a Group 1 carcinogen, is a significant environmental and public health issue. The toxicity of arsenic varies with its chemical form. For example, inorganic species like arsenite (AsO_3_^3−^) and arsenate (AsO_4_^3−^) are generally more toxic than organoarsenic compounds. However, some organoarsenic species exhibit higher toxicity than inorganic species. Therefore, the precise quantification and speciation of arsenic is necessary. Chromatographic techniques, particularly liquid chromatography coupled with inductively coupled plasma mass spectrometry (LC-ICP-MS), are widely used for arsenic speciation owing to their high sensitivity and accuracy. Gas chromatography-mass spectrometry (GC-MS) is another effective technique for detecting arsenic species after derivatization. In addition to chromatographic methods, more straightforward and cost-effective techniques are available for inorganic arsenic speciation. These include adsorption techniques, colorimetric assays such as the molybdenum blue method, hydride generation reactions, and voltammetry. Emerging technologies, such as microfluidic and electrochemical devices, enable rapid and portable analysis, facilitating in situ detection of arsenite and arsenate in environmental samples. While LC-ICP-MS remains the gold standard for comprehensive arsenic speciation, other advanced technologies provide a practical, rapid, and cost-effective approach.

**Graphical Abstract:**

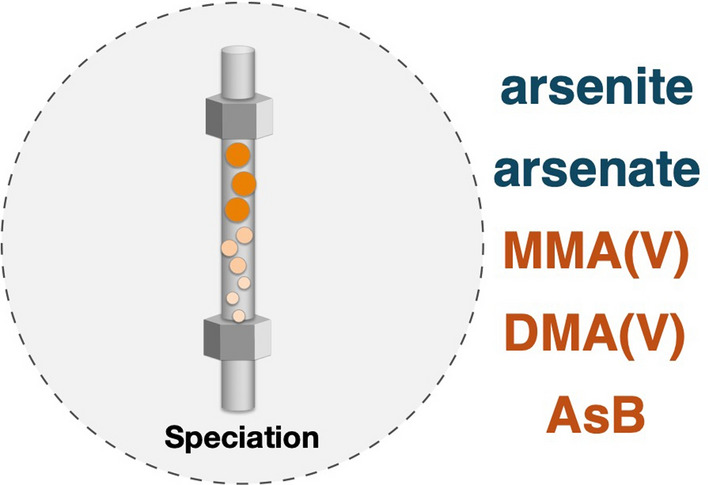

## Introduction

**Fig. 1:**
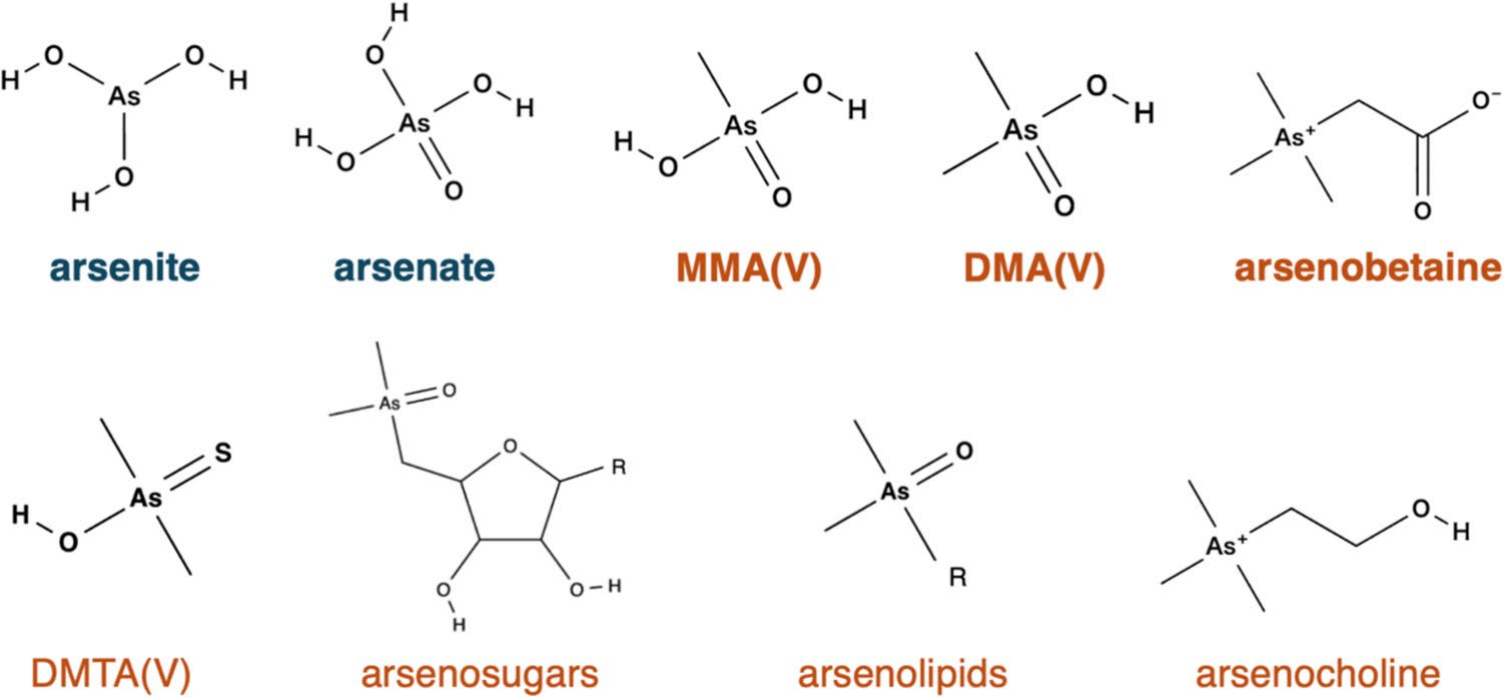
Structure of naturally occurring arsenic species. Bold: five major species frequently measured by LC-ICP-MS, blue: inorganic arsenic species, orange: organic arsenic species

Arsenic is one of the most toxic elements and is classified as a Group 1 carcinogen by the International Agency for Research on Cancer (IARC). Arsenic pollution remains a global environmental threat [1, 2], causing severe health problems worldwide [3]. Khute et al. recently reported elevated arsenic concentrations in leafy vegetables in India, suggesting arsenic contamination in the soil and groundwater [4]. In addition, Bangladesh is also known as one of the most severely affected areas by arsenic contamination [5]. In these Asian countries, people are exposed to arsenic by drinking highly contaminated groundwater drawn from wells [5, 6]. Even outside Asia, drinking water samples in various regions have shown arsenic concentrations exceeding the World Health Organization (WHO) provisional guideline value of 10 µg L^−1^ [7]. The toxicity of arsenic depends significantly on its chemical form. Inorganic arsenic species, such as arsenite (AsO_3_^3−^) and arsenate (AsO_4_^3−^), are generally more toxic than organoarsenic compounds, such as monomethylarsonic acid (MMA(V)), dimethylarsinic acid (DMA(V)), arsenobetaine, arsenocholine, and arsenosugar (Fig. 1). However, some organoarsenic compounds, such as monomethylarsonous acid (MMA(III)) and dimethylarsinous acid (DMA(III)), which are metabolic intermediates of arsenic methylation, exhibit higher toxicity than inorganic arsenic species [8]. Therefore, both alkylation and valency state are crucial factors in determining the toxicity of arsenic compounds. Moreover, dimethylthioarsinic acid (DMTA(V)), a pentavalent organoarsenic compound generated through DMA(V) metabolism by *Escherichia coli* in the human intestinal environment, exhibits carcinogenic potential [9, 10]. Thioarsenicals, including DMTA(V), are newly identified arsenic metabolites in the urine of various animals, however, little is known about their biosynthesis and toxicity profiles [11]. Given the significant implications for health and safety, conducting quantification and speciation analysis of arsenic in food, environmental, and biological samples is vital. This mini-review summarizes recent advances in speciation techniques.

## Speciation of arsenic by chromatography technique

**Fig. 2:**
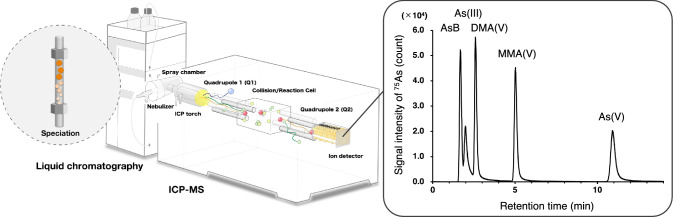
Schematic illustration of liquid chromatography hyphenated with inductively coupled plasma mass spectrometry for the speciation of major arsenic species

Liquid chromatography (LC) techniques, including size exclusion, hydrophilic interaction, reverse-phase, and ion exchange chromatography, are suitable for the separation of inorganic and organic arsenic species. Arsenic is usually detected using inductively coupled plasma mass spectrometry (ICP-MS), inductively coupled plasma atomic emission spectrometry (ICP-AES), atomic absorption spectrometry (AAS), or atomic fluorescence spectrometry (AFS). Among these, LC hyphenated to ICP-MS is the most frequently used analytical technique for arsenic speciation (Fig. 2a), supported by reliable certified reference materials of arsenic [12]. The fact that diet is one of the main routes of arsenic exposure has led to many studies on arsenic concentration and speciation in food. Arsenic contamination is a major concern for seafood [13, 14], rice [15], and vegetables [4], However, speciation analysis has recently been applied to various products, including edible insects [16] and milk [17]. In these food samples, five arsenic species are typically measured separately by liquid chromatography coupled with inductively coupled plasma mass spectrometry (LC-ICP-MS): arsenobetaine, DMA(V), MMA(V), arsenite, and arsenate. In some cases, arsenocholine [18] and arsenosugar [14] are also included in the analysis. Although various types of chromatography can be used for arsenic speciation, anion exchange chromatography is most frequently used in food analysis. In anion exchange chromatography, the mobile phase typically includes (NH_4_)_2_HPO_4_ or (NH_4_)_2_CO_3_ [13–15, 17], and approximately 1–3% of methanol [13, 14, 18] is sometimes added to enhance peak resolution and reduce measurement time. Most studies achieve speciation analysis within 15 min, with sufficient peak resolution for the aforementioned arsenic species.

Although arsenic is a monoisotopic element, its detection by ICP-MS is challenging due to relatively high ionization potential of 9.82 eV. Furthermore, mass spectrometric interferences by polyatomic ions such as ^40^Ar^35^Cl^+^, ^40^Ca^35^Cl^+^, and ^40^Ca^16^O(^1^H_3_^16^O)^+^ and doubly charged ions such as ^150^Nd^2+^ and ^150^Sm^2+^, can hinder precise arsenic quantification. However, recent advances in collision/reaction cell technology have significantly mitigated these interferences. The use of helium or hydrogen as a collision gas can preferentially reduce signals from isobaric interferences [19]. Additionally, oxygen as a reaction gas enables the detection of arsenic as ^75^As^16^O^+^ in ICP-MS/MS, effectively removing mass spectrometric interferences [20]. Although the limit of detection (LOD) varies among arsenic species due to differences in column recovery and ionization efficiency, most LOD values achieved by LC-ICP-MS are below 1.0 μg L^−1^ for arsenic [15, 17].

Liquid chromatography-mass spectrometry (LC-MS) coupled with anion exchange chromatography detects arsenic species based on their molecular weight, with a slightly higher LOD compared to LC-ICP-MS [21]. Therefore, LC-MS, when used solely or in combination with LC-ICP-MS, allows for the identification of unknown arsenic species and the speciation of target arsenic species [21, 22].

In addition to LC, gas chromatography-mass spectrometry (GC-MS) can separately measure organoarsenic species such as MMA(V) and DMA(V), as well as inorganic arsenic after derivatization. Because GC-MS can detect molecular ions, the identification and speciation of various chemical forms of arsenic can be accomplished without certified reference materials. Thiols (e.g., methylthioglycolate (TGM) and ethylthioglycolate (TGE)) and dithiols (e.g., 1,3-propanedithiol (PDT), 1,5-pentanedithiol (PDTe), and 2,3-dimercapto-1-propanol (BAL)) are used for the derivatization of MMA(V) and DMA(V) to produce volatile and thermally stable compounds. Although inorganic arsenic species can also be converted into volatile sulfur derivatives, arsenate is reduced to arsenite with TGM and TGE, forming derivatives [23, 24]. Therefore, the speciation of arsenite and arsenate using these reagents is difficult. In contrast, BAL reacts more rapidly with arsenite than arsenate at room temperature [25]. To obtain derivatives of arsenate with BAL, arsenate is reduced to arsenite with tin chloride and potassium iodide before derivatization [26]. Alternatively, arsenate is reacted with BAL at a high temperature of approximately 50 to 60℃ [25]. It is possible to perform a stepwise extraction of the derivatives of arsenite and arsenate with BAL using reductants (i.e., tin chloride and potassium iodide) and a monolithic silica spin column [26], enabling the speciation of inorganic arsenic species by GC-MS. In GC-MS analysis following derivatization, the LOD is higher (e.g., 6 μg L^−1^ for DMA(V) and 14 μg L^−1^ for MMA(V) [24]) compared to that in LC-ICP-MS.

## Speciation of inorganic arsenic

Although ADMET (i.e., absorption, distribution, metabolism, excretion, and toxicity) varies with the chemical form of arsenic, the majority of arsenic compounds found in the environment are inorganic forms [27]. Therefore, the determination and speciation of inorganic arsenic (i.e., arsenite and arsenate) in environmental samples are extensively performed. Compared with chromatography, the speciation of inorganic arsenic is easy to perform and inexpensive, and devices for in situ analysis have been developed.

Inorganic arsenic species can be selectively extracted using adsorption techniques, such as solid-phase microextraction, liquid-phase microextraction, and others [28]. Owing to their capability for surface modification and their high surface-to-volume ratio, nanomaterials have been used as sorbents for metal ions, enabling metal-specific quantitative extraction. As regards arsenic, silica-coated magnetic nanoparticles with differently modified surfaces can separately collect arsenite and arsenate, namely, nanoparticles modified with 3-mercaptopropyltrimethoxysilane for arsenite and 3-aminopropyltriethoxysilane for arsenate [29]. Mai et al. reported that reduced graphene oxide/magnetite (rGO/Fe_3_O_4_) nanocomposites efficiently removed arsenate [30]. These nanoparticle-based arsenic removal techniques are summarized elsewhere [31]. The methyl-esterified egg-shell membrane has also shown specificity for arsenate adsorption over a wide pH range, facilitating inorganic arsenic speciation [32]. These studies indicate that nanoparticles and other solid sorbents effectively adsorb inorganic arsenic. Speciation can be achieved through selective adsorption or extraction procedures.

Inorganic arsenic species are typically measured using the molybdenum blue method [33], which is suitable for determining phosphate and arsenate through spectrophotometry. A commercially available detection kit for phosphate that utilizes this method is available. It can also be employed to analyze arsenic species in environmental water samples under optimized conditions [34–36]. Interference by phosphate ions is estimated by adjusting the acidity of the solution, as phosphate selectively reacts with molybdate in highly acidic conditions [37], and by reducing arsenate to arsenite to avoid the reaction with molybdate [35]. Furthermore, total inorganic arsenic content is determined by oxidizing arsenite to arsenate. Therefore, the speciation of arsenite and arsenate is fundamentally possible. Spectrophotometer, commonly used to detect arseno-molybdate complex, is less sensitive than mass spectrometry. Dhar et al. reported the LOD was greater than 7 μg L^−1^ using a single-beam spectrophotometer [34].

The hydride generation reaction is also applicable for detecting inorganic arsenic. Volatile arsine (AsH_3_) can be directly introduced into ICP-MS, AAS, or AFS. Additionally, arsine produced by reducing arsenite can be detected through colorimetry or spectrometry. A yellow precipitate is produced by the well-known Gutzeit reaction of arsine with mercury chloride [38]. As arsine is easily transformed into other oxidized arsenic forms, ferric ions can be reduced to ferrous ions by arsine, forming a red complex with phenanthroline (Fe(II)-1,10-phenanthroline), which is detected by a colorimetric sensor using a polymeric thin film [39]. Similarly, the reduction of ionic gold (Au^3+^) to pink to violet metallic Au nanoparticles (Au^0^) can also be used for arsenic detection [40]. Bonacci et al. developed a hydride generation microfluidic paper-based analytical device (µPAD) that detects arsenic through the formation of Au nanoparticles [40]. In this device, only arsenite is reduced in the presence of hydrochloric acid. Still, both arsenite and arsenate are reduced to arsine in the presence of sulfuric acid, suggesting the potential for speciation of inorganic arsenic species. In these studies, arsenic is indirectly detected through the reduction of other substances using a spectrophotometer. Consequently, the reported LOD value ranges from 410 to 1350 μg L^−1^ [39, 40], which is higher than that achieved with other techniques.

Inorganic arsenic species can be determined using several voltammetric techniques, and speciation is achieved by obtaining both the total arsenic content and the arsenite or arsenate content. Bullen et al. reported an arsenate-specific chemisorbent material named “ImpAs” that can separately collect arsenite from arsenate. They determined the contents of both arsenite and total inorganic arsenic by anodic stripping voltammetry, accomplishing the speciation of inorganic arsenic species in water samples [41]. Pungjunun et al. developed a microfluidic electrochemical device that can simultaneously determine the contents of arsenite and total inorganic arsenic [42]. This device can determine the content of total inorganic arsenic obtained by reducing arsenate with thioglycolic acid. Henze et al. demonstrated the possibility of differentiating arsenite and arsenate during the reduction step in cathodic stripping voltammetry by altering the composition of the supporting electrolyte [43]. Arsenite content is selectively determined using a mannitol-free electrolyte, whereas total inorganic arsenic content is assessed using a supporting electrolyte containing mannitol after the oxidation of arsenite to arsenate.

In summary, the speciation of arsenic species is primarily achieved using LC-ICP-MS techniques. Furthermore, microfluidic devices and other emerging technologies for quantifying inorganic arsenic species allow for rapid quantification and speciation analysis of environmental samples, potentially facilitating in situ analysis.

## Data Availability

This review article is based on previously published data, which are publicly available and cited in the references. No new data were generated or analyzed in this study.
